# COVID-19 vaccination in patients with long QT syndrome

**DOI:** 10.1016/j.hroo.2022.07.011

**Published:** 2022-08-03

**Authors:** Cheng-I. Wu, Peter J. Schwartz, Michael J. Ackerman, Arthur A.M. Wilde

**Affiliations:** ∗ Amsterdam UMC, University of Amsterdam, Heart Center; Department of Clinical and Experimental Cardiology, Amsterdam Cardiovascular Sciences, Amsterdam, The Netherlands; † Heart Rhythm Center, Division of Cardiology, Department of Medicine, Taipei Veterans General Hospital, Taipei, Taiwan; ‡ Center for Cardiac Arrhythmias of Genetic Origin and Laboratory of Cardiovascular Genetics, Istituto Auxologico Italiano, IRCCS, Milan, Italy; § European Reference Network (ERN) GUARD-Heart; ‖ Departments of Cardiovascular Medicine (Division of Heart Rhythm Services and the Windland Smith Rice Genetic Heart Rhythm Clinic), Pediatric and Adolescent Medicine (Division of Pediatric Cardiology), and Molecular Pharmacology & Experimental Therapeutics (Windland Smith Rice Sudden Death Genomics Laboratory), Mayo Clinic, Rochester, Minnesota

**Keywords:** COVID-19, Long QT syndrome, SARS-CoV-2, Vaccinations, Inherited cardiac arrhythmias

## Abstract

Patients with long QT syndrome (LQTS) face potential threats from COVID-19 vaccination. Fever is one of the issues that is not uncommon after vaccination, and it usually takes place within 2 days. In particular, patients with type 2 LQTS based on trafficking-deficient variants are probably vulnerable to arrhythmogenicity under febrile conditions. Furthermore, myocarditis is one of the rare complications that is possibly associated with acquired QT prolongation and puts patients with LQTS at risk of life-threatening arrhythmia. Moreover, postural orthostatic tachycardia syndrome is another rare condition that, perhaps, poses LQTS patients susceptible to life-threatening arrhythmia when QT interval does not shorten optimally during tachycardia. In this review, we recommended prudent measurements to beneficially reduce the risk for patients with LQTS when vaccination or booster doses are eligible.

**Key Findings**

▪ Patients with type 2 long QT syndrome (LQTS) are probably vulnerable to arrhythmogenicity under febrile conditions.
▪ In the extremely rare event that an LQTS patient experiences myocarditis following either the infection itself or the vaccination, monitoring the QTc seems a reasonable precaution.
▪ If patients with type 1 LQTS have any sign of postural orthostatic tachycardia syndrome after COVID-19 vaccination, monitoring the electrocardiogram may be reasonable in the initial phase.

## Introduction

Ever since the outbreak of the severe acute respiratory syndrome coronavirus 2 (SARS-CoV-2)–COVID-19 pandemic, patients with inherited arrhythmia syndromes have faced potential threats either from COVID-19 itself or from the potentially beneficial medication,^1^^,^^2^ such as hydroxychloroquine and remdesivir, though some of these are no longer standard treatment.

Inherited long QT syndrome (LQTS) is a genetic heart disease caused predominantly by pathogenic variants in *KCNQ1*-encoded Kv7.1 channels (type 1 LQTS, LQT1), *KCNH2*-encoded Kv11.1 channels (type 2 LQTS, LQT2), and *SCN5A*-encoded Nav1.5 channels (type 3 LQTS).^3^, ^4^, ^5^ It is characterized by prolonged ventricular repolarization and an increased risk for torsadogenic syncope/seizures, sudden cardiac arrest, and sudden cardiac death.^4^^,^^5^ Therefore, monitoring the change of QTc could provide adequate management proportionate to the risk of life-threatening arrhythmic events (LAEs) in circumstances of trigger factors.

In the past years, inherited arrhythmia specialists have faced frequent questions concerning the safety of COVID-19 vaccines. However, owing to the lack of disease-specific data, the answers to these questions are usually based on the safety data of available vaccines. In this brief review, we attempt to provide data specific to LQTS that may help either general physicians or inherited arrhythmia specialists manage the question in several aspects.

## COVID-19 and LQTS

**Figure 1:**
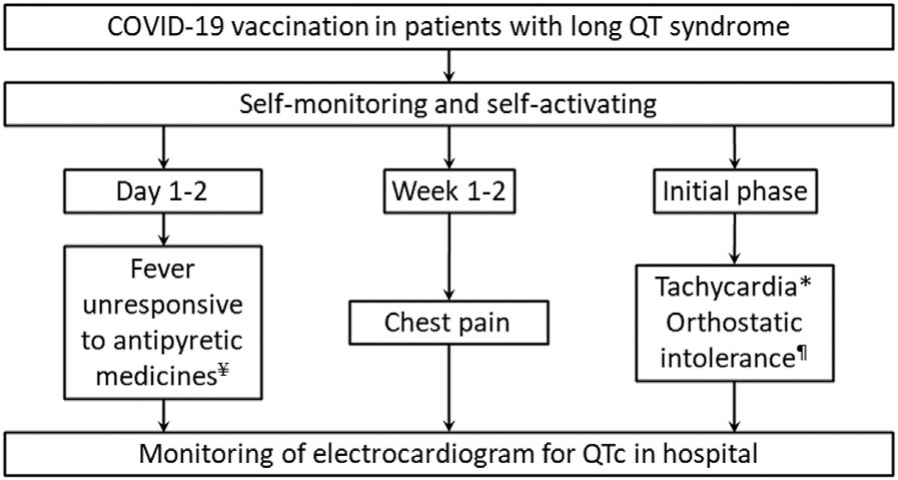
A proposed management algorithm of COVID-19 vaccination for patients with long QT syndrome (LQTS). When patients with LQTS receive COVID-19 vaccination, self-monitoring and self-activating are recommended for several conditions. Fever usually occurs within 48 hours after the vaccination; therefore, prophylaxis with antipyretic agents is recommended, especially for patients with type 2 LQTS and particular mutations (A558P and F640V). In such patients with persistent fever despite antipyretic agents, monitoring of corrected QT interval (QTc) in hospitals maybe considered. In addition, myocarditis is a rare severe complication after vaccination, and it usually takes place within 2 weeks after the vaccination and is accompanied by chest pain. Likewise, if chest pain appears and myocarditis is highly suspected, monitoring of QTc in hospitals may be considered for patients irrespective of LQTS. Postural orthostatic tachycardia syndrome (POTS) is probably another rare condition after the vaccination, and the approximate time from vaccination is still inconclusive. Patients may require QTc monitoring in the initial phase when POTS is suspected and goes along with tachycardia. ^¥^Antipyretic agents 1–2 days after vaccination. ∗An increase of heart rate (≥30 beats/min) in response to 10 minutes of head-up tilt or standing and without orthostatic hypotension (fall of blood pressure ≥30/20 mm Hg). ^¶^Orthostatic intolerance after standing or head-up tilt (eg, lightheadedness, weakness, palpitations, blurred vision, breathing difficulties, nausea, or headache).

COVID-19 itself is one of the factors to influence QTc,^6^ by either hypoxia or exaggerated immune response (Supplemental Figure 1). In setting of hypoxia, an elevated late sodium current can further increase action potential duration of ventricles to prolong QTc.^7^ The effect of cytokines seems also of importance.^8^ For example, interleukin-6 that is triggered by SARS-CoV-2 infection is likely involved in QTc prolongation by directly modulating cardiac ion channel function.^6^ An early review of the literature clearly demonstrated a significant QT prolongation in the 425–455 ms range (compared to ±400 ms in healthy volunteers; Figure 1 in reference 6) in hospitalized COVID-19 patients.^9^ With the addition of more recent studies, a comparable result was obtained (mean, 425 ms; range 399–461 ms, compared with a mean of 407 ms; range 399–432 ms in the control population). The association between the SARS-CoV-2 virus and prolonged QTc was displayed in the absence of known QT-prolonging medications (Supplemental Figure 2 and references). In addition, there is in vitro evidence that the SARS-CoV-2-associated spike protein prolongs the action potential duration of human induced pluripotent stem cell–derived cardiomyocytes (Ackerman, unpublished data).

However, despite the awareness that SARS-CoV-2-mediated COVID-19 is an acquired form of QT prolongation, there has been no signal of increased LAEs among our patients with LQTS (3 major centers, data unpublished). During now more than 2 years of careful global monitoring in our 3 centers and through the Sudden Arrhythmia Death Syndromes Foundation, there is no suggestion that the LQTS-associated cardiac event rate among diagnosed and treated LQTS patients is any different than before SARS-CoV-2 virus. Further, patients with genetic electrical heart diseases like LQTS are not at high risk for adverse COVID-19 outcomes just because of their genetic substrate. So, our present recommendations align with the overarching LQTS-relevant preventative measures like avoiding QT-prolonging medications whenever feasible (www.crediblemeds.org) and are not unique to the COVID-19 pandemic.

## COVID-19 vaccines and LQTS

Whether COVID-19 vaccines directly impact the QTc is still unclear. Current worldwide COVID-19 vaccines are composed of 2 major categories: mRNA-based vaccines and viral vectored vaccines.^10^ Pfizer-BioNTech COVID-19 vaccine and Moderna COVID-19 vaccine are mRNA-based vaccines, and Johnson & Johnson (Janssen) COVID-19 vaccine and AstraZeneca COVID-19 vaccine are viral vectored vaccines. mRNA-based vaccines contain the RNA that has been modified to allow the evasion of innate immune responses in hosts, which is encapsulated in a PEGylated lipid nanoparticle to help cell entry. Viral vectored vaccines make use of recombinant viruses that are revised to encode antigens obtained from the target pathogen to infect host cells. Both types of vaccines evoke a cellular immune response by antigens that are produced by host cells and are presented on human leukocyte antigen class 1.^11^ These vaccines help people defend against the invasion of SARS-CoV-2, but people also stand a potential risk of adverse reactions that are mediated by the immune response.

## The influence of fever on LQTS

Fever is one of the potential issues with both COVID-19 itself and the COVID-19 vaccines^12^ aiming to prevent the SARS-CoV-2 infection or minimize its consequences if infected postvaccination. Therefore, paying attention to body temperatures is necessary because febrile status could unmask the electrocardiographic (ECG) manifestations and make patients with electrical heart diseases vulnerable to LAEs.^1^ Brugada syndrome (BrS) is a well-known example. Kokawa and colleagues^13^ recently reported a BrS case getting febrile-associated LAEs after COVID-19 vaccination, and an implanted cardioverter-defibrillator intervention was needed. Based on this case, prophylaxis with antipyretic agents and a fever self-monitoring is recommended for BrS patients within the first 2 days after vaccination.^12^ In contrast, the effect of fever has potentially much less impact in patients with LQTS. However, patients with LQT2 and particular LQT2-causative variants (ie, A558P, F640V) might be a possible exception.^14^ These are trafficking-deficient variants, and co-expression with the wild-type protein causes a dominant negative effect. At higher temperature, the increase in wild-type current was less in the presence of the mutant than in the absence.^14^ The experimental data align with the clinical data, including QTc prolongation during higher body temperature and fever-triggered arrhythmias.^14^ Whether all trafficking-deficient variants, which form the majority of pathogenic *KCNH2* variants,^15^ have a similar effect is unknown but would not be unexpected. Lim and colleagues^16^ also have reported a case of a patient with LQT2 who experienced fever-induced polymorphic ventricular tachycardia. Hence, adequate precautions after COVID-19 vaccination may be prudent for patients with LQT2 as well (Figure 1).

## Myocarditis and QT prolongation

Among our 3 dedicated LQTS specialty centers that oversee the care of nearly 5000 patients, we have not had a single case of clinically diagnosed myocarditis in any of our patients with LQTS. However, myocarditis is one of the rare severe complications following COVID-19 vaccination, particularly associated with mRNA-based vaccines in males aged 16–29 years.^17^^,^^18^ Furthermore, all patients had chest pain, and symptoms began around 2.4 days (range, 1–16 days) after the vaccination. Although most cases were related to mRNA-based vaccines, especially after the second dose,^19^ the myocarditis also occurred with viral vectored vaccines.^20^ Patients with vaccine-related myocarditis had a longer QTc than patients without myocarditis (444 ms vs 425 ms).^21^ A cohort of 40 patients with acute non-COVID-19-related or vaccine-related myocarditis found that a fulminant course of myocarditis was not only associated with a longer QTc (483 ± 70 ms vs 412 ± 33 ms; *P* value .016) on admission, it also results in a rapid decline of cardiac function and fatal ventricular arrhythmias, in which QT prolongation will further put patients at risk of arrhythmogenic events.^22^ Based on these data, myocarditis probably contributes to QT prolongation, and patients with LQTS may be at increased risk if they develop postvaccination myocarditis. If an LQTS patient experiences myocarditis, even though an extremely rare event, monitoring the QTc seems a reasonable precaution (Figure 1).

## Postural orthostatic tachycardia syndrome and its possible effect on LQTS

Postural orthostatic tachycardia syndrome (POTS) is another rare condition that may result from COVID-19 vaccination, supported by at least 2 case reports.^23^^,^^24^ It is a disorder characterized by a noticeable increase of heart rate (≥30 beats/min) in response to 10 minutes of head-up tilt without orthostatic hypotension (fall of blood pressure ≥30/20 mm Hg) and with symptoms of orthostatic intolerance after standing or head-up tilt (eg, lightheadedness, weakness, palpitations, blurred vision, breathing difficulties, nausea, or headache).^25^ The majority of cases with POTS involved females at a young age.^26^^,^^27^ The underlying mechanism of POTS after COVID-19 vaccines is still unclear. Either autoantibodies against α1-adrenergic receptors in the cardiovascular system or diminished response to angiotensin II contributes to the impairment of vasoconstriction and induced postural tachycardia.^3^^,^^28^^,^^29^

During tachycardia the QT interval should adapt. I_Ks_ is managed by K_V_7.1, in which the α-subunit is encoded by *KCNQ1*. The genetic deficit in this gene is related to LQT1 and makes QT interval fail to shorten adequately after tachycardia.^30^ Therefore, if patients with LQT1 have any sign of POTS after COVID-19 vaccination, monitoring the ECG may be reasonable in the initial phase (Figure 1). Optimal therapeutic options for POTS have been described in detail elsewhere,^27^ though no medicine is currently specific when this problem is happening to patients with LQTS. We know that β-blocker plays an important role in LQTS, and adherence to this medicine has been emphasized.^4^ However, it is also an agent to worsen orthostatic intolerance in POTS.^27^ Whether this should lead to change in medication (dose) in LQTS patients with COVID-associated POTS is unknown.

## Conclusion

Taken together, it has been quite encouraging to see that after nearly 2 years of this COVID-19 pandemic and now more than 1 year of COVID-19 vaccinations, a properly diagnosed and well-treated patient with LQTS is NOT at any greater risk of LQTS-triggered cardiac events when either infected with SARS-CoV-2 or vaccinated for COVID-19. Nevertheless, akin to an “ounce of prevention is worth a pound of cure,” recognizing that the virus can precipitate acquired QT prolongation and that fever (from either the infection or the vaccine) could be an LQTS agitator, particularly in LQT2 patients, these simple preventative measures (lowering fever, avoiding QT-prolonging drugs, and monitoring the QTc) seem prudent. To be sure, the risk-benefit calculus clearly favors that patients with LQTS or any other genetic heart disease get vaccinated if age-eligible and then get boosted when time-eligible.
