# Supplementary material for: COVID-19 vaccination in patients with long QT syndrome
Source: Heart Rhythm O2. 2022 Aug 3;3(6Part A):706–9. doi: 10.1016/j.hroo.2022.07.011 (PMC9345651; doi:10.1016/j.hroo.2022.07.011)
Supplement: Supplemental material [file mmc1.docx]

**Supplemental material**

**Figure 1**





**Figure 1. QTc, COVID-19, and the optimal management.** COVID-19 can influence QTc, either by hypoxia or exaggerated immune response. In addition, viruses can alter the ion transport in host cells and induce abnormal Ca^2+^ handling and downregulation of K^+^ channels to affect QTc.^1,2^ Comorbidities, such as diabetes, adiposity, or metabolic syndrome present in hospitalized COVID-19 patients, may add on the QT interval prolongation.^3^ Therefore, timely monitoring QTc, promptly correcting the electrolytes, stopping QTc-prolonged drugs, and avoiding bradycardia could be of importance when patients with long QT syndrome get infected by SARS-CoV-2.

**Figure 2**





**Figure 2. Baseline QTc in the population with COVID-19 and the control group.**According to the literature in the past two years, plenty of studies have demonstrated the QTc change caused by COVID-19 during the disease period. Although many of them addressed the effect of Chloroquine or other drugs on QTc, these publications also provided the values of QTc before the treatment. Therefore, based on these various studies^4-56^, we noticed that the baseline QTc of the patients with COVID-19 was longer than that of the control population before initiation of the therapy. Error bars indicate standard deviations of each separate study. The filled cycles represent the mean value of QTc in each study. The horizontal black lines are the mean values of QTc of the two groups, 425ms (range, 399–462ms) for the COVID-19 group and 407ms (range, 399–432ms) for the control group.

**References**

1. Tse G, Yeo JM, Chan YW, Lai ET, Yan BP. What is the arrhythmic substrate in viral myocarditis? Insights from clinical and animal studies. Front Physiol 2016;7:308.

2. Hover S, Foster B, Barr JN, Mankouri J. Viral dependence on cellular ion channels–an emerging anti-viral target? J Gen Virol 2017;98:345-351.

3. Park B, Lee YJ. Metabolic syndrome and its components as risk factors for prolonged corrected QT interval in apparently healthy Korean men and women. J Clin Lipidol 2018;12:1298-1304.

4. Chorin E, Wadhwani L, Magnani S, et al. QT interval prolongation and torsade de pointes in patients with COVID-19 treated with hydroxychloroquine/azithromycin. Heart Rhythm. 2020 Sep;17:1425-1433.

5. Ocak M, Tascanov MB, Yurt NŞ, Yurt YC. A new predictor for indicating clinical severity and prognosis in COVID-19 patients: Frontal QRS-T angle. Am J Emerg Med 2021;50:631-635.

6. Pepe M, Napoli G, Brindicci G, et al. Prognostic value of 12-leads admission electrocardiogram in low-risk patients hospitalized for Covid-19. Minerva Med 2021. Online ahead of print. <https://doi.org/10.23736/S0026-4806.21.07894-0>

7. Gumilang RA, Siswanto, Anggraeni VY, Trisnawati I, Budiono E, Hartopo AB. QT interval and repolarization dispersion changes during the administration of hydroxychloroquine/chloroquine with/without azithromycin in early COVID 19 pandemic: A prospective observational study from two academic hospitals in Indonesia. J Arrhythm 2021;37:1184-1195.

8. Minguito-Carazo C, Echarte-Morales J, Benito-González T, et al. QT interval monitoring with handheld heart rhythm ECG device in COVID-19 patients. Glob Heart 2021;16:42.

9. Zhao W, Gandhi N, Affas S, Szpunar S, Mesiha N, Saravolatz L. Predicting QT interval prolongation in patients diagnosed with the 2019 novel coronavirus infection. Ann Noninvasive Electrocardiol 2021;26:e12853.

10. Bakhshaliyev N, Özdemir R. The impact of hydroxychloroquine-azithromycin combination on Tpeak-to-end and Tpeak-to-end/QT ratio during a short treatment course. Ann Noninvasive Electrocardiol 2021;26:e12846.

11. Shaghee F, Nafakhi H, Alareedh M, Nafakhi A, Al-Buthabhak K. ECG markers of malignant arrhythmias and in-hospital outcome of COVID-19 pneumonia. J Arrhythm 2021;37:426–31.

12. Akhtar Z, Gallagher MM, Yap YG, et al. Prolonged QT predicts prognosis in COVID-19. Pacing Clin Electrophysiol 2021;44:875-882.

13. Changal K, Paternite D, Mack S, Veria S, Bashir R, Patel M, et al. Coronavirus disease 2019 (COVID-19) and QTc prolongation. BMC Cardiovasc Disord 2021;21:158.

14. Haghjoo M, Golipra R, Kheirkhah J, et al. Effect of COVID-19 medications on corrected QT interval and induction of torsade de pointes: Results of a multicenter national survey. Int J Clin Pract 2021;75:e14182.

15. Uğurlu Ilgın B, Akbulut Koyuncu İM, Kızıltunç E. Effect of triple antimicrobial therapy on electrocardiography parameters in patients with mild-to-moderate coronavirus disease 2019. Anatol J Cardiol 2021;25:184-190.

16. Singh A, Akbar MS, McElroy D, et al. The electrocardiographic manifestations and derangements of 2019 novel coronavirus disease (COVID-19). Indian Pacing Electrophysiol J 2021;21:156-161.

17. Matteucci A, Massaro G, Sergi D, et al. Electrocardiographic modifications and cardiac involvement in COVID-19 patients: results from an Italian cohort. J Cardiovasc Med (Hagerstown) 2021;22:190-196.

18. O'Connell TF, Bradley CJ, Abbas AE, et al. Hydroxychloroquine/azithromycin therapy and QT prolongation in hospitalized patients with COVID-19. JACC Clin Electrophysiol 2021;7:16-25.

19. Becker ML, Snijders D, van Gemeren CW, Kingma HJ, van Lelyveld SFL, Giezen TJ. QTc prolongation in COVID-19 patients using chloroquine. Cardiovasc Toxicol 2021;21:314-321.

20. Echarte-Morales J, Minguito-Carazo C, Del Castillo-García S, et al. Effect of hydroxychloroquine, azithromycin and lopinavir/ritonavir on the QT corrected interval in patients with COVID-19. J Electrocardiol 2021;64:30-35.

21. El Ouarradi A, Abdeladim S, Oualim S, et al. Hydroxychloroquine and azithromycin as a treatment of COVID-19: electrocardiogram variability. J Saudi Heart Assoc 2020;32:350-357.

22. Özdemir İH, Özlek B, Özen MB, Gündüz R, Çetin N, Bilge AR. Hydroxychloroquine/azithromycin treatment, QT interval and ventricular arrhythmias in hospitalised patients with COVID-19. Int J Clin Pract 2021;75:e13896.

23. Gunay S, Caliskan S, Sigirli D, Sahin E. Ventricular repolarization indexes in patients treated with hydroxychloroquine - azithromycin combination for COVID-19. Bratisl Lek Listy 2020;121:817-821.

24. Lanza GA, De Vita A, Ravenna SE, et al. Electrocardiographic findings at presentation and clinical outcome in patients with SARS-CoV-2 infection. Europace 2021;23:123-129.

25. Huang HD, Jneid H, Aziz M, et al. Safety and effectiveness of hydroxychloroquine and azithromycin combination therapy for treatment of hospitalized patients with COVID-19: A propensity-matched study. Cardiol Ther 2020;9:523-534.

26. Bernardini A, Ciconte G, Negro G, et al. Assessing QT interval in COVID-19 patients:safety of hydroxychloroquine-azithromycin combination regimen. Int J Cardiol 2021;324:242-248.

27. Braunstein ED, Reynbakh O, Krumerman A, Di Biase L, Ferrick KJ. Inpatient cardiac monitoring using a patch-based mobile cardiac telemetry system during the COVID-19 pandemic. J Cardiovasc Electrophysiol 2020;31:2803-2811.

28. Bakhshaliyev N, Uluganyan M, Enhos A, Karacop E, Ozdemir R. The effect of 5-day course of hydroxychloroquine and azithromycin combination on QT interval in non-ICU COVID19(+) patients. J Electrocardiol 2020;62:59-64.

29. Yenerçağ M, Arslan U, Doğduş M, et al. Evaluation of electrocardiographic ventricular repolarization variables in patients with newly diagnosed COVID-19. J Electrocardiol 2020;62:5-9.

30. Lazzerini PE, Laghi-Pasini F, Acampa M, Boutjdir M, Leopoldo Capecchi P. IL-6 (Interleukin 6) blockade and heart rate corrected QT interval prolongation in COVID-19. Circ Arrhythm Electrophysiol 2020;13:e008791.

31. Hooks M, Bart B, Vardeny O, Westanmo A, Adabag S. Effects of hydroxychloroquine treatment on QT interval. Heart Rhythm 2020;17:1930-1935.

32. Maraj I, Hummel JP, Taoutel R, et al. Incidence and determinants of QT interval prolongation in COVID-19 patients treated with hydroxychloroquine and azithromycin. J Cardiovasc Electrophysiol 2020;31:1904-1907.

33. Ramireddy A, Chugh H, Reinier K, et al. Experience with hydroxychloroquine and azithromycin in the coronavirus disease 2019 pandemic: Implications for QT interval monitoring. J Am Heart Assoc 2020;9:e017144.

34. Habibzadeh P, Moghadami M, Lankarani KB. The effect of potential therapeutic agents on QT interval in patients with COVID-19 Infection: The importance of close monitoring and correction of electrolytes. Med Hypotheses 2020;143:109847.

35. Moschini L, Loffi M, Regazzoni V, Di Tano G, Gherbesi E, Danzi GB. Effects on QT interval of hydroxychloroquine associated with ritonavir/darunavir or azithromycin in patients with SARS-CoV-2 infection. Heart Vessels 2021;36:115-120.

36. Tuncer T, Karaci M, Boga A, Durmaz H, Guven S. QT interval evaluation associated with the use of hydroxychloroquine with combined use of azithromycin among hospitalised children positive for coronavirus disease 2019. Cardiol Young 2020;30:1482-1485.

37. Alqarawi W, Birnie DH, Golian M, et al. The clinical utility of continuous QT interval monitoring in patients admitted with COVID-19 compared with standard of care: A Prospective Cohort Study. CJC Open 2020;2:592-598.

38. Mercuro NJ, Yen CF, Shim DJ, et al. Risk of QT interval prolongation associated with use of hydroxychloroquine with or without concomitant azithromycin among hospitalized patients testing positive for coronavirus disease 2019 (COVID-19). JAMA Cardiol 2020;5:1036-1041.

39. Bessière F, Roccia H, Delinière A, et al. Assessment of QT intervals in a case series of patients with coronavirus disease 2019 (COVID-19) infection treated with hydroxychloroquine alone or in combination with azithromycin in an intensive care unit. JAMA Cardiol 2020;5:1067-1069.

40. Gasperetti A, Biffi M, Duru F, et al. Arrhythmic safety of hydroxychloroquine in COVID-19 patients from different clinical settings. Europace 2020;22:1855-1863.

41. Fteiha B, Karameh H, Kurd R, et al. QTc prolongation among hydroxychloroquine sulphate-treated COVID-19 patients: An observational study. Int J Clin Pract 2021;75:e13767.

42. Brüggemann RJ, Moes DJAR, van Rhee KP, et al. Chloroquine for treatment of COVID-19 results in subtherapeutic exposure and prolonged QTc intervals. Int J Antimicrob Agents 2021;57:106293.

43. Gulletta S, Della Bella P, Pannone L, et al. QTc interval prolongation, inflammation, and mortality in patients with COVID-19. J Interv Card Electrophysiol 2021:1–8.

44. Ding J, Liu W, Guan H, et al. Corrected QT interval in hospitalized patients with coronavirus disease 2019: Focus on drugs therapy. Medicine (Baltimore) 2021;100:e26538.

45. Roccia H, Argaud L, Le Goic M, Guérin C, Cour M. Electrocardiogram monitoring in the prone position in coronavirus disease 2019 acute respiratory distress syndrome. Eur J Cardiovasc Nurs 2021;20:792-796.

46. Cipriani A, Zorzi A, Ceccato D, et al. Arrhythmic profile and 24-hour QT interval variability in COVID-19 patients treated with hydroxychloroquine and azithromycin. Int J Cardiol 2020;316:280-284.

47. Çap M, Bilge Ö, Işık F, et al. The effect of favipiravir on QTc interval in patients hospitalized with coronavirus disease 2019. J Electrocardiol 2020;63:115-119.

48. Vink AS, Neumann B, Lieve KVV, et al. Determination and interpretation of the QT interval. Circulation. 2018 Nov 20;138:2345-2358.

49. Mzayek F, Deng H, Mather FJ, et al. Randomized dose-ranging controlled trial of AQ-13, a candidate antimalarial, and chloroquine in healthy volunteers. PLoS Clin Trials 2007;2:e6.

50. Murat B, Akgun H, Akarsu M, Ozmen A, Murat S. The impact of hydroxychloroquine and azithromycin on the corrected qt interval in patients with the novel Coronavirus disease 2019. Rev Assoc Med Bras (1992) 2021;67:979-984.

51. Farré N, Mojón D, Llagostera M, et al. Prolonged QT interval in SARS-CoV-2 infection: prevalence and prognosis. J Clin Med 2020;9:2712.

52. Rubin GA, Desai AD, Chai Z, et al. Cardiac corrected QT interval changes among patients treated for COVID-19 infection during the early phase of the pandemic. JAMA Netw Open 2021;4:e216842.

53. Etheridge SP, Asaki SY. COVID-19 infection and corrected QT interval prolongation-collateral damage from our newest enemy. JAMA Netw Open 2021;4:e217192.

54. Sinkeler FS, Berger FA, Muntinga HJ, Jansen MMPM. The risk of QTc-interval prolongation in COVID-19 patients treated with chloroquine. Neth Heart J 2020;28:418-423.

55. Saleh M, Gabriels J, Chang D, et al. Effect of chloroquine, hydroxychloroquine, and azithromycin on the corrected QT interval in patients with SARS-CoV-2 infection. Circ Arrhythm Electrophysiol 2020;13:e008662.

56. Borba MGS, Val FFA, Sampaio VS, et al. Effect of high vs low doses of chloroquine diphosphate as adjunctive therapy for patients hospitalized with severe acute respiratory syndrome coronavirus 2 (SARS-CoV-2) infection: A Randomized Clinical Trial. JAMA Netw Open 2020;3:e208857.
